# Evaluating a Theoretically Informed and Cocreated Mobile Health Educational Intervention for First-Time Hearing Aid Users: Qualitative Interview Study

**DOI:** 10.2196/17193

**Published:** 2020-08-05

**Authors:** David W Maidment, Rachel Heyes, Rachel Gomez, Neil S Coulson, Heather Wharrad, Melanie A Ferguson

**Affiliations:** 1 School of Sport, Exercise and Health Sciences Loughborough University Loughborough United Kingdom; 2 National Institute for Health Research Nottingham Biomedical Research Centre Nottingham United Kingdom; 3 Hearing Sciences, Division of Clinical Neuroscience School of Medicine University of Nottingham Nottingham United Kingdom; 4 Nottingham University Hospitals National Health Service Trust Nottingham United Kingdom; 5 Division of Rehabilitation, Aging and Wellbeing School of Medicine University of Nottingham Nottingham United Kingdom; 6 School of Health Sciences University of Nottingham Nottingham United Kingdom; 7 National Acoustic Laboratories Sydney Australia

**Keywords:** hearing loss, hearing aids, telemedicine, behavioral medicine, qualitative research, mobile phone

## Abstract

**Background:**

Adults living with hearing loss have highly variable knowledge of hearing aids, resulting in suboptimal use or nonuse. This issue can be addressed by the provision of high-quality educational resources.

**Objective:**

This study aims to assess the everyday experiences of first-time hearing aid users when using a newly developed, theoretically informed cocreated mobile health (mHealth) educational intervention called m2Hear. This intervention aims to deliver greater opportunities for individualization and interactivity compared with our previously developed multimedia intervention, C2Hear.

**Methods:**

A total of 16 first-time hearing aid users trialed m2Hear for a period of 10-weeks in their everyday lives, after which individual semistructured interviews were completed. The data were analyzed using an established deductive thematic analysis procedure underpinned by the Capability, Opportunity, Motivation-Behavior model. The model stipulates that to engage in a target behavior, an individual must have physical and psychological capability, physical and social opportunity, and automatic and reflective motivation.

**Results:**

Capability—m2Hear was viewed as a concise and comprehensive resource, suitable for a range of digital literacy skills. It was stated that m2Hear could be conveniently reused to provide useful reminders that facilitate knowledge of hearing aids and communication. Opportunity—m2Hear was simple and straightforward to use, enabling greater individualization and independence. The availability of m2Hear via mobile technologies also improved accessibility. Motivation—m2Hear provided greater support and reassurance, improving confidence and empowering users to self-manage their hearing loss.

**Conclusions:**

Overall, this qualitative study suggests that m2Hear supports first-time hearing aid users to successfully self-manage their hearing loss postfitting. Furthermore, this study demonstrates the utility of employing a combined theoretical and ecologically valid approach in the development of mHealth educational resources to meet the individual self-management needs of adults living with hearing loss.

**Trial Registration:**

ClinicalTrials.gov NCT03136718; https://clinicaltrials.gov/ct2/show/NCT03136718

## Introduction

### Background

Chronic health conditions, including hearing loss, are a leading cause of morbidity and mortality worldwide [1]. Empowering patients with long-term conditions to manage their own health can improve outcomes as well as quality of life [2]. Nevertheless, supporting patients to successfully self-manage their health is complex and multifaceted [3]. In the context of hearing loss, hearing aids are the primary clinical intervention strategy. As such, self-management support in aural rehabilitation should facilitate knowledge of hearing loss and hearing aids, hearing aid handling and communication skills, monitoring the development of and solving new problems, psychosocial well-being, and collaborative decision making with hearing health care professionals [4-6].

However, knowledge about hearing loss and hearing aids is often poor. First-time users, for instance, experience difficulties using their hearing aids because they struggle to remember all of the information given to them by their audiologist at the time of fitting [7,8]. Similarly, hearing aid handling skills in existing hearing aid users are highly variable, ranging from poor to excellent [9]. As a result, hearing aids are often used suboptimally or not at all, with estimates of nonuse varying from 3% to 24% [10]. Unmanaged hearing loss results in persistent psychosocial difficulties that can lead to social withdrawal and isolation for both individuals and their frequent communication partners [11,12]. Although audiological counseling post hearing aid fitting aims to address suboptimal use and nonuse of hearing aids, information in clinical settings is typically delivered verbally. Consequently, most of the information provided to patients is forgotten or retained incorrectly [13-15]. However, this difficulty can be overcome through the provision of supplementary educational support.

### Our Original Multimedia Intervention: C2Hear

We previously developed a home-delivered educational intervention for first-time hearing aid users based on the concept of reusable learning objects (RLOs) [16,17], which aimed to improve hearing aid use. RLOs are interactive *chunks* of multimedia learning that contain highly visual components, such as animations, video clips, and patient testimonials. The RLOs that we have developed cover a range of topics, prioritized by hearing health care professionals, and include both practical (eg, *how to insert hearing aids*; *hearing aid care*) and psychosocial (eg, *what to expect when wearing hearing aids*; *communication tactics*) aspects of the adult aural rehabilitation process [16]. A registered randomized controlled trial (RCT) involving 203 first-time hearing aid users demonstrated that, in comparison to standard care, the RLOs were clinically effective; users of the RLOs demonstrated superior practical hearing aid handling skills, better knowledge of hearing aids and communication, and greater hearing aid use in those who did not wear hearing aids all of the time [17]. Additionally, in a further clinically registered RCT, the RLOs were also shown to significantly improve self-efficacy for hearing aids [18]. Following the original RCT, the RLOs were refined based on participant feedback and are now called C2Hear [19,20]. In November 2015, C2Hear was made freely available via YouTube [19] and currently averages around 7000 views per month, with over 250,000 views globally. In 2019, a dedicated website for people living with hearing loss, frequent communication partners, and health care professionals was launched [20].

Although C2Hear has been shown to provide a range of benefits, there are several shortcomings. C2Hear was originally developed for a DVD-based platform because research at the time of development suggested that this format would be most accessible to the first-time hearing aid user age group (70-74 years) [21]. However, a DVD mode of delivery limited opportunities for individualization and interactivity. Additionally, the RLOs were between 5 and 8 min in duration, with some participants in the original RCT reporting that the RLOs were too long. Finally, the *one-size-fits-all* approach made it difficult to locate personally relevant information with ease [17]. Mobile health (mHealth) interventions, defined as health practices that are supported by mobile devices [22], could address these limitations. Specifically, mHealth interventions delivered via smartphone technologies (eg, smartphones, tablets, wearables) have been shown in other chronic health care domains, such as diabetes and asthma, to provide a platform that is both accessible and engaging, promoting greater self-management [23,24]. Consequently, we redeveloped C2Hear into an mHealth intervention (ie, m2Hear) that aims to meet the specific informational needs of first-time hearing aid users [25,26].

### Development of an mHealth Intervention: m2Hear

It has been recognized for some time that, to be effective, health-related behavior change interventions should be underpinned by the appropriate behavioral theory [27]. Popular theories applied within the field of audiology include the Health Belief Model [28], Theory of Planned Behaviour [29], and Transtheoretical Model [30]. However, these models have been widely criticized because they often fail to explain variations in complex human behavior [31]. Coulson et al [31] subsequently argued that the Capability, Opportunity, Motivation-Behavior (COM-B) model, a contemporary supratheory of behavior change, is better suited to understanding and describing behavior. The COM-B model proposes that for an individual to engage in a specific health-related behavior (B), they must have physical and psychological capability (C), physical and social opportunity (O), and automatic and reflective motivation (M). A more detailed understanding of capability, opportunity, and motivation can be further derived from the theoretical domains framework (TDF), which consists of a number of different constructs (Multimedia Appendix 1) that are necessary to bring about behavior change [32]. In combination, the COM-B model and TDF can be used to identify the essential components (or *active ingredients*) that should be included in an intervention to facilitate the target behavior.

**Figure 1:**
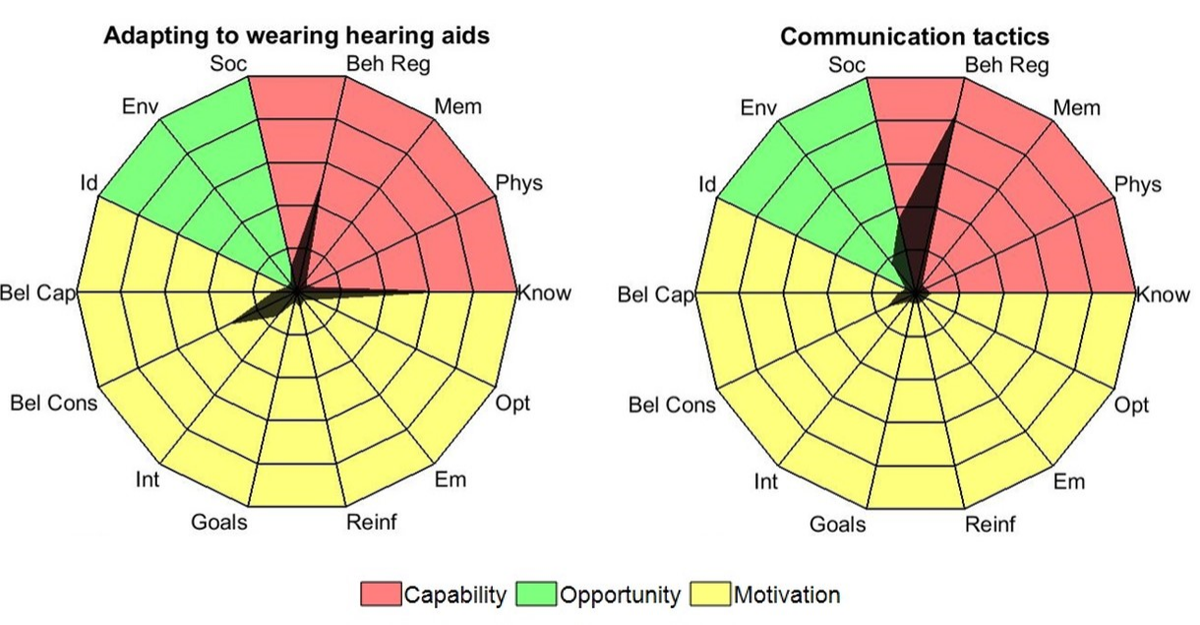
Radar chart showing the proportion of time the theoretical domains framework (TDF) factors were included in the communication tactics and adapting to wearing your hearing aids C2Hear reusable learning objects (RLOs). Percentages are plotted for the 14 TDF factors on individual axes. Concentric grid lines connecting axes increase in 20% increments, from 0% (center point) to 100% (outer edge). Each data point has been connected to form the black shaded area.

Consequently, the redevelopment of C2Hear into m2Hear was theoretically grounded, whereby the COM-B model and TDF were used to identify the components of the original C2Hear RLOs that facilitate the intended target behavior (ie, hearing aid use) [33]. Overall, we found that all RLOs consisted of multiple TDF components associated with capability (ie, knowledge, skills, memory and decision processes, behavioral regulation). However, different RLOs covered a broad range of domains relating to opportunity and motivation. For example, the *communication tactics* RLO included a high proportion of content related to opportunity, such as social influences and environmental context, whereas the *adapting to wearing your hearing aids* RLO contained content specific to motivation, such as beliefs about consequences, intentions, and goals (Figure 1).

Further to this theoretical underpinning, we employed an ecologically valid approach, whereby the original C2Hear RLOs were repurposed into 42 shorter mobile-enhanced RLOs (mRLOs). Each mRLO was designed to be a small *chunk* of learning, each with a mean duration of about 1 min (range 20 seconds to 1 min and 56 seconds). To ensure that m2Hear met the needs of the end user, a think-aloud technique was used to label each mRLO. This technique is an established observational method [34], which has been widely used in health research to develop and evaluate digital interventions [35]. We completed the think-aloud interviews with existing hearing aid users who watched the mRLOs and concurrently described their views on the content in their own words. Using an established inductive thematic analysis procedure [36], each mRLO was then labeled in accordance with data generated from participants. For example, the *what to expect when wearing hearing aids* RLO was divided into the following 2 mRLOs: (1) *What can I expect when wearing hearing aids for the first time?* and (2) *How do I get used to wearing my hearing aids?* These short mRLOs, along with the option to select the appropriate earmold coupling (open fit or custom earmold) and 5 higher-level categories corresponding to the likely need along the patient’s journey post hearing aid fitting (eg, *using your hearing aids*; *looking after your hearing aids*), aimed to provide individualized learning opportunities, whereby the user could decide what they wanted to view according to their own needs and preferences.

**Figure 2:**
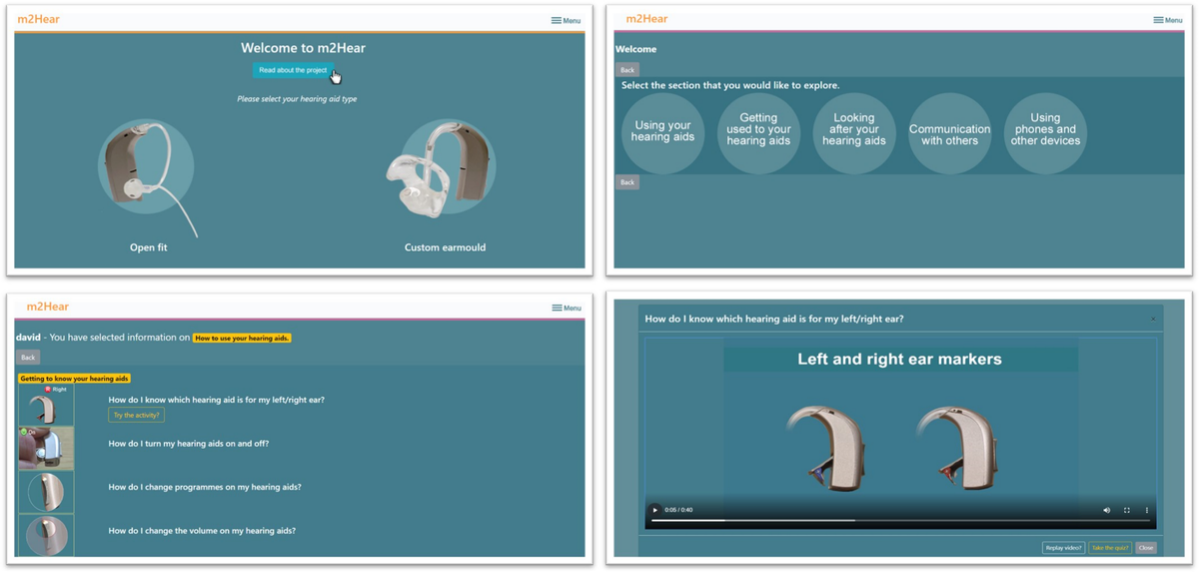
Screenshots of the m2Hear intervention, which is a freely available web-based intervention.

In addition to individualization, the mHealth intervention also enabled users to actively engage in a range of optional learning activities and quizzes to further enhance an individual’s learning potential. For example, a *drag-and-drop* activity was developed that accompanied the mRLOs *How do I put my hearing aids in?* and *How do I take my hearing aids out?* This activity required users to place images in the correct order to reinforce the mRLO learning objectives, namely, how to correctly insert and remove the earmold and hearing aid. Both individualized and interactive elements were incorporated into the design of the mobile platform for delivery of the intervention, a process that was iterative and followed a user-centered and participatory design approach. The final m2Hear intervention is a freely available web-based intervention [37]; see also Figure 2 for screenshots.

### Study Aims

Following the development of m2Hear, a clinically registered study [38] assessed the feasibility of the intervention in naïve first-time hearing aid users using a mixed methods approach. In this paper, we aimed to present the results of the qualitative evaluation of this study. Specific aims were as follows:

1. Gain an in-depth insight into the views of first-time hearing aid users toward the barriers and facilitators of using m2Hear in everyday life underpinned by the COM-B model and TDF.
2. Compare barriers and facilitators between m2Hear and C2Hear to assess whether the individualized and interactive elements incorporated in the newly developed m2Hear intervention result in greater patient benefits.

## Methods

### Participants

A total of 16 participants were recruited from the Nottingham Adult Audiology Service, Nottingham University Hospitals National Health Service (NHS) Trust. A purposive sampling strategy was used, whereby participants met the following inclusion criteria: (1) adults aged ≥18 years, (2) adults who had never worn hearing aids, (3) adults who were familiar with smartphone technologies, and (4) adults who had a good understanding of the English language to understand the mRLO content. Exclusion criteria included those who were unable to use m2Hear unassisted due to cognitive decline or dementia, determined via a self- or familial report. The demographic information of the sample is provided in Table 1. Overall, participants presented with mild-to-moderate hearing loss and self-reported as *competent* users of digital technologies [21].

**Table 1:** Demographic information of participants who trialed either m2Hear alone or both m2Hear and C2Hear.

Demographic characteristics		m2Hear only	m2Hear and C2Hear	Overall
**Gender, n (%)**				
	Female	3 (30)	3 (50)	6 (37)
	Male	7 (70)	3 (50)	10 (63)
**Age (years)**				
	Mean (SD)	67.70 (11.01)	70.67 (17.53)	68.81 (13.32)
	Range	46-81	39-85	39-85
**Better ear pure-tone average (0.25-4 kHz; dB HL)**				
	Mean (SD)	26.90 (5.55)	33.50 (8.34)	28.79 (6.85)
	Range	17-34	25-45	17-45
**Self-reported digital technology competency, n (%)**				
	Beginner	0 (0)	2 (33)	2 (12)
	Competent	10 (100)	4 (67)	14 (88)

### Procedure

Potentially eligible participants were invited to participate in the study during their hearing assessment appointment at the Nottingham Adult Audiology Service. Interested patients attended an initial study session at the National Institute for Health Research (NIHR) Nottingham Biomedical Research Centre (BRC). This session was completed shortly after participants had received their hearing aids (mean 5.06, SD 4.28 days postfitting). During this session, participants were shown how to use m2Hear and were asked to use it in their everyday lives as and when required. They were encouraged to use as much of the content that they felt was relevant to them. A subgroup of participants (n=6) was also asked to use the original C2Hear intervention via YouTube, which they were asked to compare to their use of m2Hear.

Following a period of independent use (mean 10.82, SD 0.70 weeks), participants attended a second study session at the NIHR Nottingham BRC. All participants were interviewed by the lead author (DM). The interview schedules were flexible due to the semistructured design of the interviews, although the core content remained the same (Multimedia Appendices 2 and 3). The interviews were conducted face-to-face in a quiet room and lasted for approximately 1 hour. Each interview was audio-recorded and transcribed verbatim.

All participants were each paid a nominal inconvenience allowance and travel expenses. The study was approved by the NHS Health Research Authority, East of England–Cambridgeshire and Hertfordshire Research Ethics Committee, and Nottingham University Hospitals NHS Trust Research and Innovation Department.

### Data Analysis

NVivo 10 software (QSR International) was used to organize and support the analysis of the semistructured interview data. Anonymized identification codes were assigned to each participant (eg, M060, M061, etc). The data were analyzed using the thematic analysis procedure by Braun and Clarke [36], which consists of specific analytical phases: data familiarization, generating initial codes, searching for themes, reviewing themes, and defining and naming themes. The analysis was deductive (or theoretical), as themes were derived from the components of the TDF, which each link to a specific aspect of the COM-B model [32]. In this study, themes were considered in relation to the barriers and facilitators that impacted the use of m2Hear in everyday life. The process of combining or redefining the codes led to the generation of initial themes. Overarching themes stemmed from the TDF, while inductive subthemes were devised by the first author. Subthemes were defined as something important about the data that captured important information about the research aims and represented repeated patterns of response or meaning that were prevalent (ie, reported by several participants) across the entire data set [36]. For rigor, coding comparison [39] was undertaken to ensure that the interpretation of the data was not limited to the perspective of the first author. Specifically, a second author (RH) independently coded 6 (37%) of the transcripts and formulated potential themes. Any discrepancies were discussed, and an agreement was made regarding which codes should be applied. The themes were refined and defined through re-analysis of the data and discussions among coauthors (DM and RH).

## Results

Theoretical domains that were and were not coded against the TDF are shown in Table 2. A summary of the subthemes classified according to the TDF is also provided in Table 3. Each domain was mapped onto the corresponding determinants of the target behavior (ie, use of m2Hear) in accordance with the COM-B model (Multimedia Appendix 1).

**Table 2:** Theoretical domains that were coded against the theoretical domains framework and those that were not.

COM-B^a^, TDF^b^		Coded?
**Capability**		
	Knowledge	No
	Physical skills	Yes
	Memory, attention, and decision processes	Yes
	Behavioral regulation	No
**Opportunity**		
	Social influences	Yes
	Environmental context	Yes
**Motivation**		
	Social and professional role and identity	No
	Beliefs about capabilities	Yes
	Beliefs about consequences	No
	Intentions	No
	Goals	Yes
	Reinforcement	No
	Emotion	No
	Optimism	No

^a^COM-B: Capability, Opportunity, Motivation-Behavior.

^b^TDF: theoretical domains framework.

**Table 3:** A summary of subthemes generated in relation to the Capability, Opportunity, Motivation-Behavior model and theoretical domains framework.

COM-B^a^, TDF^b^		Subtheme
**Capability**		
	Physical skills	Digital literacy skills
	Memory, attention, and decision processes	Conciseness
	Memory, attention, and decision processes	Repeated use to aid memory
**Opportunity**		
	Social influences	Independence and autonomy
	Social influences	Relevance to communication partners
	Social influences	Requirement of audiological input
	Environmental context	Convenience and portability
	Environmental context	Ease of use and individualization
**Motivation**		
	Beliefs about capabilities	Learning and understanding
	Beliefs about capabilities	Reassurance and self-efficacy
	Goals	Improving hearing aid handling and communication skills

^a^COM-B: Capability, Opportunity, Motivation-Behavior.

^b^TDF: theoretical domains framework.

### Capability

#### Digital Literacy Skills

Having sufficient digital literacy skills was considered essential for users to successfully access and use m2Hear. For example, M067 commented, “I could use it, and then my Dad could use it, so…there’s no sort of barriers in it. It’s open to everyone to use.” Some thought that m2Hear might be difficult to use for older people who lack digital skills. M061 remarked, “I think if you’re computer-savvy…it could be a benefit...for other people, I’m just thinking that the older age group, I think, will struggle.” As a potential solution, friends or family could support the use of m2Hear:

Somebody else who hasn’t got that confidence or the capability or the technology to do it, needs to talk to somebody…If they’ve got that reference, they’ve got a daughter or a son...they can be assisted by their own family.

M065

Nevertheless, given the ubiquitous nature of smartphone technologies, digital literacy was considered to be less of a concern:

The only people who won’t have [m2Hear] available to them all the time are those who haven’t got a smartphone, a tablet or a computer. I would say they’re going to be non-existent, very shortly. I mean, even Granny’s got a smartphone now.

M065

#### Conciseness

The short mRLOs enabled participants to locate personally relevant information with ease:

I think the value is that you can just go to the subjects you want. For bits that you're not interested in, well, what's the point of going to that? What you want is information, and to be able to go straight to that bit of information, I think, is a valuable part of [m2Hear].

M064

The succinct mRLOs also encouraged access and re-engagement, “They’re concise and logical, and that’s why I went back to them a couple of times” (M065). Furthermore, the mRLOs were a good use of time and effort: M062 said, “It meant best use of my time. I didn’t have to spend ages getting bored and wondering where it was, I could clearly see what it was I needed.” The concise content also held participants’ interest, which improved information retention:

It’s better because you only have a certain amount of attention span, and I think because they are only like a minute, that’s about right…there’s so much more information in longer ones, whereas if it’s a little bit of information it makes it easier to take in.

M067

Taken together, the mRLOs were concise and subsequently viewed favorably.

#### Repeated Use to Aid Memory

Although participants reported that they initially decided to view all mRLOs within the first week, they would regularly reuse m2Hear throughout the 10-week study period to improve their knowledge and understanding of specific topics. For example, participants consistently commented that they would rewatch the mRLOs for troubleshooting advice, particularly when they experienced a problem with their hearing aid or communication:

I’d click on the “communicate with others” one, and “looking after my hearing aid”. I’d click on those two if I’d had a bad day about something, so I’d click on there just to have a look, and then make sure I was looking after them properly. Just to refresh what I should do with them.

M067

It was consistently reported that m2Hear supplemented and expanded upon the information provided by the audiologist during their hearing aid fitting appointment, as well as providing useful reminders of the information they had been given:

Although the audiologist had probably given me all the right information, you don’t store it. You don’t remember it because there’s so much coming in…Because I’d got [m2Hear], I could go back, and I could go and check on something.

M062

Participants commented that it was often difficult to retain all the information provided during audiological appointments and that re-using m2Hear during the study period provided a means to refresh their memory, without feeling overwhelmed or confused:

You can only take in so much at any one time…It’s nice to have a reference to go back to it, just to check it on your own…The information is there if you need it. That’s what a [m2Hear] is all about.

M065

In addition, the quizzes and interactive features also ensured that the content of the mRLOs were successfully retained and remembered, “Yes, it was like a little test to work on my memory. Even though I was watching the [mRLO] I was probably not watching it, whereas a quiz it was making me remember stuff” (M067). Nevertheless, some participants stated that they did not think it necessary for them to reuse m2Hear once they had viewed all the mRLOs, as they understood the information and were confident using their hearing aids:

Well, in the first instance of course it was very good because it told me about the hearing aid. After a while, no, I don't think there's much more you need to know.

M068

### Opportunity

#### Independence and Autonomy

One of the main reported advantages of m2Hear was that it enabled participants to self-manage their hearing loss. This was summarized by M060:

The thing is that everyone is responsible for their own wellbeing. You shouldn’t rely on other people. For me, I don’t feel that I should be relying on other people. Other people are there in a desperate need, which is why [m2Hear] is good. As I keep saying, it’s a prop.

Participants stated that managing their hearing loss independently was crucial to their self-esteem:

I haven’t got to rely on anyone else. I haven’t got to ask someone else; I don’t like asking people…I’d rather just be able to do it myself...If I’m constantly asking people, I just feel helpless, and I don’t like that.

M067

In addition, participants were often reluctant to telephone and arrange an appointment with an audiologist due to limited health care resources and stated that m2Hear empowered them to be more self-sufficient:

I think [m2Hear] is very useful. I mean, in audiology, they tell you things and that, but the limited time. Yes, it just seems a useful reference instead of having to keep phoning someone up or go back… You're straight there for as many times as you want it.

M064

#### Relevance to Communication Partners

m2Hear was not only relevant to people living with hearing loss, but also to frequent communication partners. Indeed, several participants stated that they shared m2Hear with friends and family members to improve their communication. For example, M060 said:

I showed my wife…to make sure that she’s aware of the subtleties of not talking to me in a different room. That if she’s talking to me in a different room then we’re back to square one.

The added value of m2Hear was that it provided an objective voice that was external to the participant’s immediate social network:

I think because it’s produced by somebody professionally, rather than me just giving my opinion or my interpretation…therefore that gave it more weight as far as he *[husband]* was concerned.

M062

However, some participants preferred to use m2Hear alone, as they did not think it would be suitable or relevant for communication partners that do not have a hearing loss, such as M069, who said, “Well, I didn’t think it was particularly important, you know, really. They were…appropriate to me, but not to anybody else.”

#### Requirement of Audiological Input

Although m2Hear facilitated self-management of hearing loss, there were occasions when it was considered necessary to seek help and advice from an audiologist. Changes in the overall hearing level, for instance, would prompt participants to arrange an audiological reassessment; “If my hearing changes I would ring audiology,” remarked M060. In addition, participants reported that they would arrange an appointment if their hearing aid was faulty or causing discomfort:

I suppose if I was having specific problems with my hearing aids and thought there was something malfunctioning or something like that, then I would ring up the [audiology department] and speak to somebody.

M062

M069 also commented that they felt it was permissible to contact audiology if they experienced difficulties they were unable to resolve themselves using m2Hear: “I think if something goes wrong, you need advice about that type of thing, then…it’s nice to know that you’ve got somebody to talk to.” On this basis, m2Hear is a useful “tool” (M062) that can be used to supplement the provision of face-to-face appointments with an audiologist.

#### Convenience and Portability

Whether participants opted to access m2Hear from a handheld device (eg, smartphone, tablet) was based on immediacy and convenience. For example, M066 said, “I had a smartphone…that's what I use all the time…It's just easier to pick up and use.” Similarly, M069 commented:

I’ve got a laptop, but I don’t use it very often. The tablet is much more convenient...it’s easier to handle. It’s smaller, and also, to be able to access the Internet, it’s almost immediate. Whereas, the laptop isn’t.

Accessing m2Hear from smartphone technologies was advantageous because of portability: “It’s transferrable isn’t it, between devices…it’s portable, everywhere, any device. It makes it interesting” (M067). Furthermore, m2Hear could be used in multiple listening situations whenever desired. For example, comparing m2Hear with written information, M066 commented, “It's more convenient to use, wherever you are. You can just get your phone out, whereas you might not have the booklet with you.” Nevertheless, some participants preferred to use a laptop or desktop computer to access m2Hear, citing that a larger screen size was necessary to optimize visual acuity: “My eyesight is going as well. So, whilst the phone, I’ve got an iPhone, is great, I’m finding if I need to look at something that I feel I get a bigger picture” (M061). Taken together, while the device used to access m2Hear varied across participants, this was often based on personal preference and convenience. Nevertheless, smartphone technologies have improved accessibility.

#### Ease of Use and Individualization

All participants commented that m2Hear was simple and straightforward to use, “It wasn’t loads of diving off into other areas and that, it was…nice and simple. There wasn’t anything complicated” (M067). It was also well-organized and structured, ensuring that information could be easily located: M062 said, “It’s very easy to get to the section you need to look at or you want to look at…you can see quite clearly which elements you need to go to. That was good.” In relation, the questions accompanying each individual mRLO facilitated navigation:

They seemed relevant to the [mRLO] you'd just watched. I think the questions really weren't difficult questions. I presume the questions were designed in mind of, “Have you got the hang of what the [mRLO] was on about?” I think, yes, the questions tease that answer out quite well.

M064

Participants also reported that the organization of m2Hear enabled greater personalization:

It means that you haven’t got to start right from the beginning again…I could just go straight to the one that I want. That’s all I want to go to, and it doesn’t hold you back, it just makes it easier to access it.

M067

For these reasons, participants reported that m2Hear was preferable to written information: M065 summarized, “In the written information you’ve got to do a whole lot of searching. [m2Hear] leads you by the nose, to be quite honest, and everything is logical about it, and it just takes a click.”

### Motivation

#### Improved Learning and Understanding

Participants commented that the mRLO content was comprehensive, useful, and relevant, which they believed facilitated their knowledge of hearing aids and communication. For example, M060 said, “I can’t think of any topics that were actually left out...It’s got everything in there that I needed to know, and it’s there for me to look back on if I forget.” However, some participants stated that they would have liked additional information to have been included, such as the physiology of the ear and hearing loss, “I’d like a bit more technical depth to it...I’d like a little bit more depth about how the hearing system works” (M063). In addition to the mRLOs, optional quizzes and interactivities were also perceived to improve participants’ learning and understanding. For example, remarking on one of the activities, M060 said:

It gives you a bit of food for thought, so you think about it again. It’s no good learning something by rote, as it were. It’s understanding it. So next time you come across that you’ve got an understanding.

However, some stated that the quizzes were not beneficial, as they understood the content of the mRLO:

In my case, I think I pretty much understood everything on the [mRLOs]. The quizzes for me were slightly irrelevant in terms of my understanding, my learning. I didn’t need them.

M060

Therefore, although some participants expressed ambivalence, most stated that they used m2Hear because they believed that both the mRLOs and interactive components would improve their knowledge of hearing aids and communication, resulting in more successful outcomes.

#### Reassurance and Self-Efficacy

Several participants reported that they regularly referred back to m2Hear as a means of support and reassurance that they were handling their hearing aids correctly:

I think it's a kind of reassurance thing...You watch it and you think, “Well, yes, I am.” I think it's just useful as a reminder, having forgotten something or so on. Yes, I think it's a useful tool, isn't it? Yes, it's a good backup, I think.

M064

Subsequently, m2Hear improved both self-efficacy for hearing aids and coping with hearing loss:

I can’t reinforce how useful I feel that [m2Hear] is. As I say, I've gone back to recap on different things...It’s just really given me the confidence...I feel that I can cope with any situation with my hearing aids now.

M060

In addition, m2Hear reduced feelings of loneliness and despair, with M060 saying:

I think when I was looking at it I was not only soaking in the information and stuff, I was thinking, well, this is something that’s relevant to a lot of people...I'm not on my own, and I've got something to help me.

Therefore, using m2Hear facilitated an optimistic outlook, empowering participants to self-manage their hearing loss.

#### Improving Hearing Aid Handling and Communication Skills

The most commonly cited reason participants used m2Hear was to improve their hearing aid handling and communication skills. m2Hear helped participants to manage their expectations and encouraged them to persevere using their hearing aids:

You expect to be able to put [hearing aids] on like you put glasses on. I did think it would work in the same way. I thought it would be instant and it’s not. [m2Hear] is a useful reminder...this isn’t going to be straightforward and you’re going to have to work at it, but the benefits will be worthwhile.

M062

Moreover, m2Hear facilitated hearing aid use, especially when experiencing communication difficulties that might otherwise result in social withdrawal. For example, M067 commented that the mRLOs:

made a difference, they helped me. I mean I wouldn’t have said to people, “I can’t hear you,” I probably would have just switched off, and sort of just not bothered probably. It did help me; it gave me a bit of a boot.

As such, m2Hear had a positive impact on hearing aid use and adherence as well as communication during the 10-week evaluation.

### Differences Between m2Hear and C2Hear

In relation to the themes identified, differences between the barriers and facilitators that impacted the use of m2Hear compared with those that impacted the use of C2Hear were also reported, whereby m2Hear was consistently viewed more positively.

#### Digital Literacy

m2Hear was considered more appropriate for any level of digital literacy compared with C2Hear when accessed via YouTube. For example, M075 stated, “[m2Hear] is presented so well that I think most people, no matter how poor their understanding is, they’d still get on well with that.” In comparison, the C2Hear YouTube channel was viewed as more difficult to use if an individual had poor digital literacy skills. When using YouTube, M078 remarked:

I was terrified in case I’d make a mistake and I ruin my computer, you see. Because I’m not very computer-savvy. So, I just looked at

m2Hear

#### Conciseness

A key difference reported between m2Hear and C2Hear was the difference in length of the mRLOs. The shorter mRLOs were preferred, as participants felt that they could retain the information more easily. For example, M075 commented, “You can assimilate the knowledge more easily and you retain it perhaps better.” A further advantage of the shorter mRLOs was that it was easier to locate personally relevant information: “m2Hear was covering a topic which was concise and to the point, so you could jump straight to the information that you wanted” (M074)*.* Conversely, participants commented that the C2Hear RLOs were lengthy in duration: “I lost interest in the [C2Hear RLOs]. I found them a bit distracting because sometimes they told you a bit too much” (M076). Nevertheless, the longer RLOs might be preferable for individuals who like to access all the information in one sitting:

Sometimes the length of the [RLOs] could be nice. When you have got all the information, it’s not so bad to watch it all from beginning to end.

M076

#### Convenience and Portability

m2Hear was more accessible than C2Hear, as it was more convenient to use with smartphone technologies. For example, M074 remarked:

[m2Hear] was smarter on my iPhone to use, the interface was more workable to me on the iPhone, whereas the C2Hear...because it was on YouTube, it kept jumping up with other clips of other hearing research which was frustrating.

A further benefit of m2Hear being easily accessed via smartphone technologies was that it could be used whenever and wherever required:

It’s easier just to use [m2Hear] when you’re out and about and you just want to sit down and have a look at it and just go through it, just to remember things more, rather than wait till I get home and just look on the laptop.

M076

Conversely, participants reported that their use of C2Hear was restricted to when they had more time available due to the length of the RLOs: M076 said, “I used to do [C2Hear] when I’d got more time, so I could watch all of them, not dip in and out of it quite so much”. Taken together, convenience and portability improved the accessibility of m2Hear compared with C2Hear.

#### Ease of Use and Individualization

An important difference between m2Hear and C2Hear was that m2Hear offered the opportunity for greater personalization. In addition, it was easier to find personally relevant information in m2Hear compared with C2Hear: “[m2Hear] is so easy, and it’s so user-friendly, and it’s so clear” (M075). C2Hear was less personalized and difficult to navigate. For example, M071 remarked, “In C2Hear you’ve got to go back, find various bits, and not quite start again, but it’s much more difficult to go back.” Nevertheless, C2Hear would have been acceptable if they had not been able to compare it to m2Hear: M071 stated, “If you hadn’t shown me [m2Hear], I’d probably have been perfectly happy with [C2Hear].” Therefore, although participants were satisfied with C2Hear, they preferred m2Hear overall.

#### Learning and Understanding

Participants consistently commented that m2Hear was more interactive than C2Hear, which reinforced the knowledge gained from each mRLO and would appeal to different learning preferences. For example, M076 remarked, “It’s a different way of learning, a different way of putting information across.” In comparison, C2Hear was considered less interactive, with some participants failing to notice that there was the opportunity to take quizzes at the end of each RLO: “I didn’t spot [the quizzes] on [C2Hear]. Probably because…the [RLO] went on too long” (M076)*.*

#### Reassurance and Self-Efficacy

Compared with C2Hear, m2Hear improved confidence and enabled greater independent use of hearing aids. M074 commented:

[m2Hear] increases my confidence for looking after my hearing aids myself without needing to go and get help...It gives me reassurance that I’m doing the right thing and that I’m not going to break them.

Participants also reported that the m2Hear interactivities provided further reassurance: “They made you think and made you realise what there was and how easy it was to get the information out” (M075). Thus, in comparison to C2Hear, m2Hear improved self-efficacy for hearing aids and communication, facilitating greater self-management of hearing loss.

## Discussion

### Principal Findings

The functionality of mHealth technologies has been shown to enable greater individualization and interactivity in multiple chronic health care domains, which has the potential to improve self-management [23,24]. In this study, we assessed the barriers and facilitators of using a newly developed mHealth educational intervention, m2Hear, designed specifically for first-time hearing aid users. We assessed the views of first-time hearing aid users toward m2Hear when used in everyday life. To gain an in-depth insight into potential barriers and facilitators, participants’ experiences were evaluated within the context of the TDF and COM-B model [40,41], which are discussed as follows.

### Capability

With regard to capability, digital literacy skills were identified as important for the usability and adherence of m2Hear. For m2Hear, participants commented that any level of skill would be sufficient in this area, given that it was relatively straightforward to use and navigate, whereas C2Hear accessed via YouTube required a high level of digital literacy. It is likely that the ease of use of m2Hear is attributable to the iterative, user-centered, and participatory design approach that was employed during the development of m2Hear [25,26]. Such an approach, which has been shown to improve usability, acceptability, and adherence of interventions [42], likely ensured that m2Hear met the specific needs of the end user. This is encouraging given that the proportion of older adults (≥55 years) who use smartphone technologies continues to increase exponentially in the typical first-time hearing aid user age group [43,44]. Thus, mobile technologies should be considered an acceptable and accessible mode of delivery for educational support throughout the hearing aid user’s journey, as digital literacy skills are becoming less of a barrier in this population.

A further theme related to capability was that m2Hear was reused throughout the 10-week study period because it provided useful reminders that expanded upon the information provided by the audiologist during the hearing aid fitting appointment. This was further facilitated by the concise duration of the mRLOs, which enabled participants to easily locate and revisit the desired information with ease. In support of these findings, existing research in the area of multimedia learning recommends dividing content into shorter learning segments to reduce cognitive load (or memory capacity), improving knowledge acquisition and long-term retention [45]. On this basis, mHealth interventions have the potential to improve the likelihood that first-time hearing aid users will acquire the necessary knowledge and skills to successfully self-manage their hearing loss (eg, improve hearing aid use and social participation).

### Opportunity

One of the most pertinent social factors identified in this study was whether participants shared m2Hear with their family and friends (ie, frequent communication partners). Although some participants felt it necessary to share m2Hear with others to improve mutual communication, others did not, citing that their hearing loss was not a concern for others who did not experience hearing difficulties. This latter finding should be addressed, given that frequent communication partners play a pivotal role in hearing loss management and communication [46]. Furthermore, hearing loss in older adults can result in continued communication difficulties, leading to social isolation and withdrawal for both the person living with hearing loss and their communication partners, termed third-party hearing disability [47].

Meeting the informational needs of communication partners has been shown to be highly beneficial [12,47-49]. For example, Barker et al [12] suggest that information and support should be offered to both individuals and their communication partners to align coping strategies and improve outcomes for both parties. Consequently, we have redeveloped and tailored the original *communication tactics* C2Hear RLO into an mRLO suitable for communication partners [26]. Specifically, we have altered the wording so that it is more generic for *others*, such as family members and the general public. Interactive components have also been incorporated, including simulated hearing losses in the presence and absence of background noise. This mRLO for communication partners and the general public is available on the web [20]. The quality, usability, relevance, and impact of the repurposed mRLO have subsequently been examined using think-aloud techniques with dyads comprising adults with hearing loss and their communication partners [26]. We found that these dyads led to greater inclusivity; the mRLO enabled greater joint working and joint responsibility, whereby both parties became jointly aware of factors that prevented and facilitated optimal communication. As a result, mHealth educational interventions that incorporate greater individualization and interactivity have the potential to improve outcomes for adults living with hearing loss and their frequent communication partners. This is also highlighted in recent recommendations published by the UK National Institute for Health and Care Excellence, which states that, in addition to the person with hearing loss, information about hearing loss and how it can be managed should also be given to family members and caregivers [50].

Another theme related to opportunity included environmental factors that promoted greater use of m2Hear such as convenience and portability. These findings reflect a key benefit of mHealth technologies, as they can be used when comfort and convenience are paramount [51]. Furthermore, in the context of learning, the perceived convenience of mobile technologies has also been shown to have a positive impact on attitudes and intentions toward using an educational intervention [52]. Other environmental factors identified included the ease of finding personally relevant information due to improved organization and navigation. This is likely attributable to the extensive iterative usability testing that was employed during the development of m2Hear [25,26]. The ability to discover relevant information independently as well as to control the pace of learning via well-indexed content has been shown to enhance learning potential [53,54]. As such, these findings lend further support for the notion that a user-centered and participatory design approach should be utilized when developing mHealth interventions so that they meet the specific educational needs of the end user.

### Motivation

Participants consistently reported that they were motivated to use m2Hear because it improved their knowledge of specific topics relating to hearing aids and communication. Supplemental interactivities further improved perceived learning and understanding, presumably through active engagement with learning materials [54,55]. Participants were also motivated to use m2Hear because it provided reassurance and increased their confidence (or self-efficacy) to use hearing aids and communicate successfully. Self-efficacy refers to a domain-specific construct associated with particular tasks, abilities, skills, or actions that are needed to achieve a certain behavior, including health-related behaviors [56]. Perceived self-efficacy is being increasingly recognized as playing a key role in the audiological rehabilitation process [57-59]. Previous studies have shown that individuals with higher levels of self-efficacy are more likely to obtain hearing aids and become successful users [60-62]. In addition, self-efficacy has also been shown to predict hearing outcomes, including satisfaction [63], and has been shown to be a modifiable factor that could be targeted to improve hearing loss self-management [6]. We have shown that C2Hear significantly increases self-efficacy for hearing aids and readiness to act, with large clinical effect sizes, compared with a printed booklet. This was shown to be highly efficacious even when delivered at the hearing assessment appointment, thus priming patients before the provision of hearing aids [18].

### Study Limitations and Future Research

There are several caveats to the design of this study that could be addressed in future research. For example, a purposive sampling strategy was employed, whereby participants were recruited based on prespecified inclusion and exclusion criteria, such as familiarity with smartphone technologies. This was necessary to ensure that participants would be able to use and access m2Hear throughout the home-based evaluation. As a result, it is perhaps unsurprising that most participants self-reported as *competent* users of digital technologies, which arguably limits the generalizability of the study findings to individuals with lower levels of self-perceived competency. Future studies could address this limitation by enhancing the representativeness of the sample in terms of digital literacy skills as well as other demographic and clinical characteristics such as age, gender, and hearing loss severity.

A further consideration is that this study used a formalized, deductive (or theory-driven) thematic analysis approach, whereby themes were underpinned by the COM-B model and TDF. The application of theories and models from health psychology in audiological rehabilitation research continues to rise [11,64-68]. However, popular models frequently used in the field of audiology (eg, the Health Belief Model [28], Theory of Planned Behaviour [29], Transtheoretical Model [30]) have been widely criticized because they fail to reliably account for variations in complex human behavior [31]. As a result, Coulson et al [31] suggested that the use of unreliable models to explain and predict hearing health behaviors should be replaced by more contemporary behavior change science, namely, the COM-B model. As such, this study adds to a growing body of literature that has utilized the COM-B model to inform adult aural rehabilitation practices [26,67,69].

### Clinical Implications

As we have argued from the outset, a key advantage of mHealth interventions is that they enable the individual to tailor the information they need as well as increase user interaction, resulting in a more patient-centered approach. Patient-centered care is widely accepted as a fundamental practice that supports an individual to be an active participant in the management of their health [70]. Critically, involving patients in their own care can result in empowerment, conceptualized as a process that enhances feelings of autonomy, control, self-efficacy, and coping [71]. The concept of empowerment was also conveyed in this study, whereby participants reported that m2Hear improved their confidence to take control and participate more fully in the management of their hearing health. It is likely that this finding stems from a combination of factors afforded by using m2Hear, including increased knowledge of hearing aids and optimal communication strategies. In support, in their qualitative assessment of patients’ perspectives of empowerment, Small et al [71] found that improved knowledge and understanding is a pertinent factor necessary to empower patients to manage long-term health conditions. Additionally, identified themes surrounding reassurance and self-efficacy suggest that using m2Hear not only benefitted psychological capability but also reflective motivation for hearing loss self-management. This suggests that m2Hear fulfills 3 cornerstones of successful hearing loss self-management: (1) enabling the acquisition of knowledge, (2) prompting actions (eg, practicing hearing aid insertion), and (3) the adoption of a positive psychological stance (ie, self-efficacy). On this basis, we advocate the widespread implementation of mHealth educational resources in adult aural rehabilitation given that they have substantial potential to facilitate patient-centered care and improve hearing health outcomes.

### Summary and Conclusions

This qualitative study provides an in-depth assessment of an mHealth educational intervention used by first-time hearing aid users in their everyday lives. Underpinned by a contemporary model of health behavior change, the COM-B model, we identified key factors that influenced intervention use. Specifically, m2Hear was viewed as a concise and comprehensive resource that is simple and straightforward to use and enables greater individualization and independence. In addition, m2Hear provides greater support and reassurance, improves confidence, and empowers users to self-manage hearing loss. On this basis, this study suggests that m2Hear can be used to supplement existing aural rehabilitation practices to support successful self-management in first-time hearing aid users. Furthermore, this study demonstrates the utility of employing theoretical and ecologically valid approaches in the development of mHealth educational resources to meet the individual needs of the end user.

## Appendix

Multimedia Appendix 1 Definitions of the theoretical domains framework (TDF).

Multimedia Appendix 2 Semistructured interview schedule for participants evaluating m2Hear only (n=10).

Multimedia Appendix 3 Semistructured interview schedule for participants evaluating both m2Hear and C2Hear (n=6).

## Abbreviations

BRC: Biomedical Research Centre
COM-B: Capability, Opportunity, Motivation-Behavior
mRLO: mobile-enhanced reusable learning object
NHS: National Health Service
NIHR: National Institute for Health Research
RCT: randomized controlled trial
RLO: reusable learning object
TDF: theoretical domains framework
