# Supplementary material for: Evaluating a Theoretically Informed and Cocreated Mobile Health Educational Intervention for First-Time Hearing Aid Users: Qualitative Interview Study
Source: JMIR Mhealth Uhealth. 2020 Aug 5;8(8):e17193. doi: 10.2196/17193 (PMC7439142; doi:10.2196/17193)
Supplement: Multimedia Appendix 1 [file mhealth_v8i8e17193_app1.docx]

| **COM-B component** | **Theoretical Domains Framework** | **Definition** |
| --- | --- | --- |
| Capability | Knowledge | An awareness of the existence of something. |
|  | Physical skills | An ability or proficiency acquired through practice. |
|  | Memory, attention & decision processes | The ability to retain information, focus selectively on aspects of the environment and choose between two or more alternatives. |
|  | Behavioural regulation | Anything aimed at managing or changing objectively observed or measured actions. |
| Opportunity | Social influences | Those interpersonal processes that can cause individuals to change their thoughts, feelings, or behaviours. |
|  | Environmental context & resources | Any circumstance of a person’s situation or environment that discourages or encourages the development of skills and abilities, independence, social competence, and adaptive behaviour. |
| Motivation | Social/professional role & identity | A coherent set of behaviours and displayed personal qualities of an individual in a social or work setting. |
|  | Beliefs about capabilities | Acceptance of the truth, reality, or validity about an ability or facility that a person can put to a constructive use. |
|  | Beliefs about consequences | Acceptance of the truth, reality, or validity about outcomes of a behaviour in a given situation. |
|  | Intentions | A conscious decision to perform a behaviour or a resolve to act in a certain way. |
|  | Goals | Mental representation of outcomes or end states that an individual wants to achieve. |
|  | Reinforcement | Increasing the probability of a response by arranging a dependent relationship, or contingency, between the response and a given stimulus. |
|  | Emotion | A complex reaction pattern, involving experiential, behavioural, and physiological elements, by which the individual attempts to deal with a personally significant matter or event |
|  | Optimism | The confidence that things will happen for the best or that desired goals will be attained. |
