# Supplementary material for: Evaluating a Theoretically Informed and Cocreated Mobile Health Educational Intervention for First-Time Hearing Aid Users: Qualitative Interview Study
Source: JMIR Mhealth Uhealth. 2020 Aug 5;8(8):e17193. doi: 10.2196/17193 (PMC7439142; doi:10.2196/17193)
Supplement: Multimedia Appendix 2 [file mhealth_v8i8e17193_app2.docx]

| **Main question** | **Optional probe questions** |
| --- | --- |
| Can you tell me how you used m2Hear? | What device(s) did you use m2Hear with? And why?  Where/when/how did you use m2Hear? Which videos? What was your approach? And why?  What were the reasons for not watching all the videos?  How often did you re-visit m2Hear? What were the reasons for doing this?  How useful were the activities/videos? If useful, what did the activities and quiz provide over and above the videos? If not useful, why?  Will you use the m2Hear in the future? How might you use m2Hear in future? |
| Tell me what you think of the m2Hear. | How useful was m2Hear (e.g. improving hearing aid use, communication, confidence, coping)? Which aspects were most useful?  What did you like? What do you think were the benefits/advantages?  What didn’t you like? What do you think were the shortcomings/disadvantages? What would you change?  What was the value of tailoring m2Hear to your specific needs and preferences?  How does the m2Hear compare with the booklet? What is the value of m2Hear compared to the written booklet? |
| Can you tell me what encouraged you to use/not use m2Hear? | Other than being in the study, what were your motivations for using m2Hear?  Did m2Hear provide access to greater information? How important was it to you to have this available? (does it matter?)  Did extra information improve your confidence and provide reassurance? If so, how?  If you had difficulties with your hearing aids and communicating, why might you refer to m2Hear? Are there any occasions when would you want to seek audiologist’s opinion instead? |
| Can you tell me if m2Hear made a difference to how you used your hearing aids? | Did you think m2Hear encouraged you to use your hearing aids? If so, why and how?  Did you feel m2Hear made you wear your hearing aids more, less, or the same compared to if you had not used m2Hear? Why?  Did you refer to m2Hear to provide answers to any questions you had about how to use your hearing aids and how to better communicate with others? Why? Why not?  What were the further benefits of using m2Hear? |
| Can you tell me whether you involved others in using m2Hear? | Did you show m2Hear to other people? If so what and why?  What were the benefits or problems in showing m2Hear to others? |
