# Supplementary material for: Evaluating a Theoretically Informed and Cocreated Mobile Health Educational Intervention for First-Time Hearing Aid Users: Qualitative Interview Study
Source: JMIR Mhealth Uhealth. 2020 Aug 5;8(8):e17193. doi: 10.2196/17193 (PMC7439142; doi:10.2196/17193)
Supplement: Multimedia Appendix 3 [file mhealth_v8i8e17193_app3.docx]

| **Main question** | **Optional probe questions** |
| --- | --- |
| Can you tell me how you used m2Hear? | What device(s) did you use m2Hear with? And why?  Where did you use m2Hear? And why?  When did you use m2Hear? Which videos?  How did you use m2Hear?  How often did you re-visit m2Hear? What were the reasons for doing this?  Did you spend more time using m2Hear compared to C2Hear? And what was the reason? |
| Can you tell me how you used C2Hear? | Was your approach different to m2Hear? Did you use the same device, in the same place, and at same time?  Did you re-visit C2Hear? Why/why not? |
| Tell me what you think of the m2Hear, and how it compared to C2Hear. | How useful was m2Hear compared to C2Hear? Which aspects were most useful? Was C2Hear more/less useful?  What did you like? What do you think were the benefits/advantages of m2Hear compared C2Hear?  What didn’t you like? What do you think were the shortcomings/disadvantages compared C2Hear?  Was there anything that m2Hear offered that was better than C2Hear?  Did you encounter any problems using m2Hear and/or C2hear?  Was there any added value of tailoring m2Hear to your specific needs and preferences compared to C2Hear?  What would you change about m2Hear? Why? |
| Which did you prefer to use, m2Hear, C2Hear, neither, or both? Why/why not? | If you encountered a problem with your hearing aids and/or communication, which did you prefer to use? Why/why not?  Would you use m2Hear, C2Hear, neither, or both in the future? Why/why not? |
| Can you tell me whether you involved other people when using m2Hear? | Did you show m2Hear C2Hear, neither, or both to other people? If so what and why?  Would you recommend m2Hear, C2Hear, neither, or both, to other people? Why/why not? |
